# The Interaction of Dried Distillers Grains With Solubles (DDGS) Type and Level on Growth Performance, Health, Texture, and Muscle-Related Gene Expression in Grass Carp (*Ctenopharyngodon idellus*)

**DOI:** 10.3389/fnut.2022.832651

**Published:** 2022-04-28

**Authors:** Fatma Ragab Abouel Azm, Fanshuang Kong, Xiaoyu Wang, Wenhuan Zhu, Haojie Yu, Xianmei Long, Qingsong Tan

**Affiliations:** ^1^Key Laboratory of Freshwater Animal Breeding, Ministry of Agriculture and Rural Affairs, Wuhan, China; ^2^College of Fisheries, Huazhong Agricultural University, Wuhan, China; ^3^Department of Animal Nutrition and Clinical Nutrition, Faculty of Veterinary Medicine, Benha University, Toukh, Egypt; ^4^Fisheries Technology Extension and Guidance Center of Wuhan, Wuhan, China; ^5^Engineering Research Center of Green Development for Conventional Aquatic Biological Industry in the Yangtze River Economic Belt, Ministry of Education, Wuhan, China

**Keywords:** DDGS source, grass carp, growth performance, muscle histology, antioxidant capacity, MRFs

## Abstract

The aim of this study was to estimate the possible synergetic effects of the two levels of dietary dried distillers grains with solubles (DDGS) from different sources (US-imported and native) on the growth, health status, muscle texture, and muscle growth-related gene expression of juvenile grass carp. Four treatments of fish were fed with 4 isonitrogenous diets, namely, native DDGS20, native DDGS30, US-imported DDGS20, and US-imported DDGS30 for 60 days. The US-imported DDGS30 group showed the better growth and feed efficiency. Additionally, we observed a significant increase in hepatopancreatic total antioxidant capacity (T-AOC) and superoxide dismutase (SOD) in native DDGS groups. Moreover, raw muscle collagen increases considerably in the US-imported DDGS30 compared with the native DDGS30 group. In comparison with the native DDGS groups, the US-imported DDGS groups showed significantly decrease in all textural properties and fiber density, while increased fiber diameter. Dietary native DDGS inclusion significantly showed the upregulation of *myog, myhc*, and *fgf6a* expression in muscle, while the downregulation of the expression of *myod and myf5*. Overall, US-imported DDGS30 had a beneficial influence on growth *via* regulating genes involved in myogenesis and hypertrophy, the formation of collagen, but had negative impacts on antioxidant capacity and cooked muscle texture.

## Introduction

Protein is considered to be the most costly component of dietary nutrients in aquaculture feeds, which has a major effect on aquatic species' growth efficiency. Due to the increasing cost of traditional protein sources, researchers have attempted to develop new low-cost protein sources to replace high-cost protein sources. Dried distillers grains with solubles (DDGS), cereal by-products of the distillation process for fuel ethanol production, are the alternative sources of protein. Apart from corn, the main source of DDGS, other starch-rich cereals, such as barley, cassava, millet, rice, wheat, and sweet potato can potentially be used to produce ethanol (1). Corn DDGS, which is characterized by high-energy, moderate protein with high available phosphorus content, is composed of 26–28% protein, ~10% crude fat (as fed-basis), and residual starch, carbohydrate (2). In addition, antinutritional factors (ANFS) that are found in other protein sources, such as trypsin inhibitors in soybean meal, gossypol in cottonseed meal, and glucosinolates in rapeseed meal, are absent in DDGS (3). However, both the nutritional value and content of corn DDGS are greatly affected by the source, grain quality, fermentation process efficiency, temperature, exposure time of the drying process, and the proportion of the solubles added (4).

China is the world's third-largest producer of DDGS, and a major user of DDGS manufactured in the United States (5). China National Grains and Oils Information Center (CNGOIC) reported that, in 2011, the average price of US-imported DDGS was 1,985 yuan per metric ton, which was 19% less than the Chinese domestic corn and 35% less than soybean meal, which indicated that US-imported DDGS is a relatively low-cost feed ingredient.

Generally, US-imported DDGS has several advantages, such as better nutrient quality and consistency, compared with the Chinese DDGS due to the variation in raw materials and different milling and fermentation processes used in the Chinese native ethanol plant (6). Due to the high humidity environment of corn production, the Chinese DDGS has a higher concentration of mycotoxin than US-imported DDGS, which can be worsened by the lack of conventional processing techniques and suitable storage facilities among small farmers (7). US-imported DDGS has lower variability in energy and nutrition, as well as a higher digestibility of lysine and phosphorus, increasing its efficiency in feed composition than Chinese DDGS (8–11). Furthermore, Chinese DDGS sources are widely variable in crude fat content with higher neutral detergent fiber (NDF) content, which indicates that the Chinese DDGS has a lower and more variable metabolizable energy (ME) than the US-imported DDGS (12). Color has become a quality indicator for DDGS sources (13). The color of DDGS is linked to a number of nutritional and physical features that are altered by the differences in manufacturing and drying methods, and US-imported DDGS has a lighter, golden color, which is thought to be a subjective signal of increased protein and amino acid digestibility and feeding value (14). In addition, US-imported DDGS are lower in cost compared with domestic Chinese sources (6).

Many endogenous factors affect fish flesh quality, such as genetic factors (15), collagen content (16), and texture (17). Furthermore, feed and feeding are extrinsic factors that also influence the quality of meat (18). Mechanical textural properties, such as hardness, chewiness, springiness, cohesiveness, and adhesiveness, are common indices used to evaluate the flesh efficiency (19). The significant relationship between fiber density and several textural parameters, such as firmness has been reported in a variety of fish species (20).

Satellite cells, also known as myoblasts, are myogenic precursor cells that participate in the processes of proliferation and differentiation that contribute to muscular development (21). *MyoD, Myf5*, myogenin (*Myog*), and *Mrf4* are myogenic regulatory factors that have an important function in fish skeletal muscle growth (22). Furthermore, myostatin (*mstn*) has a negative effect on fish development (23), whereas *fgf6* and *myhc* expression have a positive effect on fish muscle proliferation and differentiation (24).

Grass carp (*Ctenopharyngodon idellus*) is the most popularly cultured freshwater herbivore fish in China, characterized by high growth, delicious taste, and good meat quality (25). The adoption of pelleted feed is one of the major reasons for the increased grass carp production at a higher density in the fish ponds. Low costs with balanced diets are the most drastic points facing aquaculture. The protein sources in fish are important, which should be accurately determined. However, DDGS in grass carp is rarely investigated, although it is widely used in fish diets. Our previous studies have determined optimal dietary native DDGS levels to replace rapeseed meal and cottonseed meal, respectively (26, 27). However, no data are available about the effect of different types of DDGS on grass carp. A growth trial by feeding four experimental groups of juvenile grass carps with diets containing two levels (20 and 30%) and the two sources of dietary DDGS (US-imported and native) was carried out in this study. Thus, the goal of this work was to compare the effects of native and US-imported DDGS on fish growth performance, health, filet texture, and muscle growth, so as to guide the use of DDGS with optimal type and level in the diets of juvenile grass carp.

## Materials and Methods

### Diets Preparation

Chinese DDGS (Feed grade) with a color of 57.7, 8.2, and 20.6 in L^*^, a^*^, b^*^ provided by Henan Tianguan Group Co., Ltd. (Nanyang) and US-imported DDGS with a color of 67.3, 7.8, and 28.9 in L^*^, a^*^, b^*^ provided by COFCO Feed (Huanggang) Co., Ltd. were determined by the UltraScan VIS colorimeter (HunterLab, Virginia, USA). Four isonitrogenous (32% protein in dry matter) and lipid (9% in dry matter) experimental diets, namely, native DDGS20, native DDGS30, US-imported DDGS20, and US-imported DDGS30 were formulated. All ingredients and the proximate analysis of the four experimental diets are provided in Table 1. The ingredients were ground to pass through a 60-mesh sieve and then thoroughly mixed with the required soybean oil and distilled water for pelleting into 1.5 mm pellets by a laboratory pellet mill (YR008; Jiedong Gangmei Yongren Foodstuff Machinery Factory, Jiedong City, Guangdong Province, China), air-dried and then kept at −20°C until use.

**Table 1:** Ingredients and the proximate analysis of the experimental diets (% in dry matter)^a^.

**Diet**	**DDGS20 (native)**	**DDGS30 (native)**	**DDGS20 (US-imported)**	**DDGS30 (US-imported)**
**Ingredients (% DM)**				
Fish meal 63% CP	3.00	3.00	3.00	3.00
Soybean meal 44% CP	22.00	22.00	22.00	22.00
Rapeseed meal 36% CP	14.00	7.00	14.00	7.00
Cottonseed meal 49% CP	11.00	11.00	11.00	11.00
Native DDGS 26% CP	20.60	30.90	0.00	0.00
US-imported DDGS 26% CP	0.00	0.00	20.60	30.90
Wheat flour 14% CP	18.00	18.00	18.00	18.00
Soya oil	2.20	1.50	2.20	1.50
Choline chloride	0.15	0.15	0.15	0.15
Ethoxyquin	0.05	0.05	0.05	0.05
Monocalcium phosphate	1.50	1.50	1.50	1.50
Microcrystalline cellulose	5.10	2.50	5.10	2.50
Yttrium oxide	0.10	0.10	0.10	0.10
Salt	0.30	0.30	0.30	0.30
Vitamin and mineral mix^a^	1.00	1.00	1.00	1.00
Cellulose	1.00	1.00	1.00	1.00
**Proximate composition (%)**				
Dry matter	89.82	89.19	89.02	88.93
Crude protein	31.96	32.19	32.44	32.54
Crude lipid	9.06	9.05	8.95	8.94
Gross energy (kJ g^−1^)	17.58	17.65	17.38	17.53

a *Provided per IU or g kg^−1^ diet: retinol, 300,000 IU; calciferol, 100,000 IU; alpha tocopherol, 1.2 g; menadione, 0.5 g; thiamin, 0.5 g; riboflavin, 0.7 g; pyridoxine, 0.6 g; cyanocobalamin, 0.0015 g; pantothenic acid, 2.5 g; ascorbyl monophosphate,10 g; folic acid, 0.15 g; calcium-D-pantothenate, 1.8 g; magnesium, 10 g; manganese, 2 g; iron, 12 g; zinc, 5 g; copper, 0.4 g; iodine, 0.1 g; cobalt, 0.03 g; selenium, 0.01 g*.

### Experimental Fish and Feeding Management

Juvenile grass carp were obtained from Honghu Fisheries Corporation (Hubei Province, China), and were acclimated to aquaculture conditions for 2 weeks. In total, 360 fish (mean initial weight, 5.0 ± 0.2 g) were randomly divided into twelve circular fiberglass tanks (300 L water capacity each) in an indoor flow-through culture system, and each tank contained 30 fish (3 tanks per treatment). Fish were manually fed to apparent satiation, two times a day at 08:00 and 15:00 for 60 days. During the feeding cycle, the daily feed intake was recorded; the water temperature and pH were kept at 26.0 ± 0.5°C and 7.0–7.6, respectively, and the water exchange was kept at 1 L/min in each tank to keep the ammonia nitrogen below 0.02 mg/L. Constant aeration was used to keep the dissolved oxygen exceeding 5 mg/L, and a natural photoperiod regime 12L:12D was applied. All procedures were approved for laboratory animal use by the Institutional Animal Care and Use Committee of Huazhong Agricultural University.

### Sampling and Chemical Analyses

At the end of the experiment, and following 24 h fasting, all grass carp were counted and weighed collectively in each tank for the calculation of growth performance parameters, such as final body weight (FBW), specific growth rate (SGR), feeding rate (FR), and feed efficiency (FE). Five fish for each aquarium were randomly chosen to measure body length (cm) and weight (g) individually to calculate the condition factor (K). The fish viscera, hepatopancreas, and mesenteric fat were weighed to measure visceral somatic index (VSI), hepatosomatic (HSI), and mesenteric fat (MFI) indexes, respectively. Blood samples were obtained from the caudal vein, precipitated at room temperature for 30 min, then centrifuged at 3,000 × g for 15 min at 4°C, and serum was extracted then frozen at −80°C. Dorsal muscle samples were quickly stored in liquid nitrogen and then preserved at −80°C till extract RNA. Further, liver samples were stored at −80°C for enzymes activity assays. In addition, hepatopancreas and muscle samples were further fixed in 4% buffer formalin for histological analysis. Moreover, muscle samples were also taken to determine the collagen content. Another 5 fish per tank were randomly sampled and stored at −20°C for body proximate analysis.

### Analytical Methods

#### Growth Performance and Body Index

The following formulas were used to calculate:

Specific growth rate (SGR, %/day) = 100 × [ln final body weight (g) – ln initial body weight (g)]/60 (days)].

Survival rate (SR, %) = (final number of fish survived/initial number of fish stocked) × 100

Feeding rate (FR, % BW/day) = 100 × [dry feed intake (g)/[60 (days) × [(final body weight (g) + initial body weight (g))/2].

Feed efficiency (FE, %) = 100 × weight gain (g)/dry feed intake (g)

Condition factor (K, %) = 100 × final body weight (g)/(body length (cm))^3^

Hepatosomatic index (HSI, %) = 100 × final hepatopancreas weight (g)/final body weight (g)

Visceral somatic index (VSI, %) = 100 × viscera weight (g)/final body weight (g)

Mesenteric fat index (MFI, %) = 100 × mesenteric fat weight (g)/final body weight (g)

#### Chemical Analysis and Textural Properties

A chemical analysis of experimental diets, whole fish body, and dorsal muscle was conducted using standard methods (28). In brief, moisture was determined by oven drying at 105°C to constant weight; protein concentration was determined by measuring nitrogen (N × 6.25) using the Kjeldahl technique; lipid was extracted by ether using the Soxhlet technique; and ash was determined after firing at 550°C for 6 h. Direct combustion of dietary samples in an adiabatic oxygen bomb calorimeter (Parr Instruments, Moline, IL, USA, model Parr 6200) to determine the gross energy. Cooking muscle loss was measured (29). In brief, a 2–3 g muscle sample was cooked for 5 min in boiling water. After drying the surface with absorbent paper, the cooked loss was equated to weight loss during heat treatment. The cooked muscle was sliced into 1 × 1 × 1 cm pieces for texture profile study (TPA) using a texture analyzer (TA.XT plus; Stable Micro Systems, UK), equipped with a 20 mm flat-bottomed cylindrical probe. Double compression was applied during testing at a steady velocity of 1.0 mm/s to reach 35% of the initial height. The pre-test speed = 2 mm/s, post-test speed = 5 mm/s, pause time = 5 s, and the data acquisition rate = 200 points per second (pps). As defined by Bourne (30), textural parameters were calculated.

#### Serum Indexes and Liver Antioxidant Capacity

The assay kits were, respectively, used to determine the serum metabolites, such as total protein (TP), alanine aminotransferase (ALAT), aspartate aminotransferase (ASAT), cholesterol (CHO), glucose (Glu), and triglycerides. In addition, total antioxidant capacity (T-AOC), superoxide dismutase (SOD), glutathione peroxidase (GSH-Px), as well as malondialdehyde (MDA) in hepatopancreatic samples were spectrophotometrically determined using commercial kits supplied by Nanjing Jiancheng Bioengineering (China).

#### Quantitative Histological Analyses

After the fixation in 4% paraformaldehyde, dorsal muscle and hepatopancreatic samples were processed by dehydration in the graded levels of ethyl alcohol. Then, the tissue was embedded in paraffin blocks. The sections of 7 μm thickness were stained with hematoxylin and eosin (H&E) for structure observation under a light microscope. An M Shot Image Analysis System (Micro-shot, Guangzhou, China) was used to measure muscle diameter and density.

#### Real-Time Quantitative PCR (q-PCR) Analysis

Using Trizol™ reagent (Takara, Dalian, China), total RNA was isolated from muscle following the manufacturer's protocol. Total RNA quantity and quality were measured by a Nanodrop spectrophotometer and a 1.0% agarose gel electrophoresis, respectively. The PrimeScript™ RT reagent Kit (Takara, Dalian, China) was used for reverse transcription of 1 μg of total RNA to generate the cDNA following the manufacturer's protocol. All primers for the genes of interest were designed using Primer Premier 6.0 software through the National Center for Biotechnology Information (NCBI) database (Table 2). The real-time quantitative PCR (q-PCR) program was performed as described previously (27). The expression results were calculated using the 2^−ΔΔCt^ method outlined by Pfaffl (31) using β*-actin* and *ef1*α as reference genes. The value in the native DDGS20 group was assigned as an arbitrary value of 1.

**Table 2:** Primers used in real-time PCR.

**Gene name**	**Primer sequence (5'-3')**	**GenBank number**
*MyoG*	Forward: TTACGAAGGCGGCGATAACTT Reverse: TGGTGAGGAGACATGGACAGA	JQ793897
*MyoD*	Forward: ATGGAGTTGTCGGATATTCCCTTC Reverse: GCGGTCAGCGTTGGTTGTT	MG544985
*Myf5*	Forward: GTGCCTGTGCCTCATCTCCT Reverse: AATGCGTGGTTCACCTTCTTCA	GU290227
*MRF4*	Forward: TCGCTCCTGTATTGATGTTGATGA Reverse: GCTCCTGTCTCGCATTCGTT	KT899334
*MyHC*	Forward: GAC GCT CAT CAC CAC CAA CC Reverse: TGC TCC TCA CGC TGC TTC T	EU414733
*MSTN*	Forward: CTGACGCCAAGTTCCACATACA Reverse: CGACTCTGCTTCAAGTTCTTCTCT	KP719016
*fgf6a*	Forward: CGCATACGAGTCTTCCAT Reverse: CCTACGAGAACATCCAACA	MK050993
*fgf6b*	Forward: TCCAGTCCGCTTCCGAGTA Reverse: AGATGAAACCCGATGCCTACA	MK050992
*β-actin*	Forward: TATGTTGGTGACGAGGCTCA Reverse: GCAGCTCGTTGTAGAAGGTG	M25013
*EF1α*	Forward: TGACTGTGCCGTGCTGAT Reverse: CGCTGACTTCCTTGGTGATT	GQ266394

*MyoG, myogenin; MyoD, myogenic determining factor; Myf5, myogenic regulatory factor 5; MRF4, myogenic regulatory factors 4; MyHC, myosin heavy chain; MSTN, myostatin; fgf6a, fibroblast growth factor 6a; fgf6b, fibroblast growth factor 6b*.

### Statistical Analysis

Results were subjected to two-way analysis of variance (ANOVA) to determine the effect of DDGS types (native and US-imported DDGS) and levels (20 and 30%), and the interaction between DDGS types and levels, using SPSS version 19 (IBM, Armonk, NY, USA). Duncan's test is used to determine significant differences among samples. The probabilities of *p* ≤ 0.05 were considered significant. Data were expressed as mean value ± SD (*n* = 3).

## Results

### Effects of Dietary DDGS Types and Levels on Growth Performance, Feed Efficiency, and Morphological Indices

A significant interaction between DDGS types and levels occurred, represented by final body weight, specific growth rate, and feed efficiency (Table 3). At the end of the feeding trial, FBW, SGR, and FE in the US-imported DDGS30 group were significantly (*p* < 0.05) higher compared with native DDGS20, native DDGS30, and US-imported DDGS20. However, survival rate and feeding rate did not show significant differences among dietary groups. As shown in Table 4, the morphological parameters represented by K, HSI, VSI, and MFI did not alter significantly with dietary DDGS inclusion levels and types (*p* > 0.05) either.

**Table 3:** The effects of different types and levels of dietary dried distillers grains with solubles (DDGS) on the growth performance and feed utilization of juvenile grass carp^a^.

**Items**	**Diet treatments**						
	**DDGS20 (native)**	**DDGS30 (native)**	**DDGS20 (imported)**	**DDGS30 (imported)**	**Inclusion level**	**DDGS types**	**Interaction**
IBW (g)	5.37 ± 0.21	5.43 ± 0.21	5.53 ± 0.21	5.43 ± 0.15	ns	ns	ns
FBW (g)	20.20 ± 0.40^a^	21.13 ± 0.40^a^	20.70 ± 0.87^a^	24.97 ± 0.40^b^	^**^	^**^	^**^
SGR (%/d)	2.20 ± 0.10^a^	2.27 ± 0.06^a^	2.20 ± 0.10^a^	2.57 ± 0.06^b^	^**^	^*^	^*^
SR (%)	100.00	98.90 ± 1.91	100.00	98.90 ± 1.91	ns	ns	ns
FR (%/d)	2.63 ± 0.06	2.60 ± 0.00	2.70 ± 0.17	2.57 ± 0.12	ns	ns	ns
FE (%)	69.73 ± 0.15^a^	71.83 ± 0.65^a^	71.20 ± 2.00^a^	85.80 ± 1.23^b^	^**^	^**^	^**^

a *Values are means ± standard deviation (SD). Mean values with different letters are significantly different at (p < 0.05)*.

* *p ≤ 0.01;*

** *p ≤ 0.001; ns, no significance; IBW, initial body weight; FBW, final body weight; SGR, specific growth rate; SR, survival rate; FR, feeding rate; FE, feed efficiency*.

**Table 4:** The effects of different types and levels of dietary DDGS on the body index of juvenile grass carp^a^.

**Items**	**Diet treatments**						
	**DDGS20 (native)**	**DDGS30 (native)**	**DDGS20 (imported)**	**DDGS30 (imported)**	**Inclusion level**	**DDGS types**	**Interaction**
K	1.98 ± 0.05	1.89 ± 0.04	1.92 ± 0.07	1.88 ± 0.06	ns	ns	ns
HSI	1.65 ± 0.18	1.57 ± 0.08	1.61 ± 0.08	1.65 ± 0.04	ns	ns	ns
VSI	10.60 ± 0.50	10.55 ± 0.56	9.96 ± 0.64	10.29 ± 0.66	ns	ns	ns
MFI	3.43 ± 0.10	3.28 ± 0.04	3.42 ± 0.23	3.27 ± 0.09	ns	ns	ns

a *Values are means ± SD. Mean values with different letters are significantly different at (p < 0.05), ns: no significance. K, condition factor; HSI, hepatosomatic index; VSI, visceral somatic index; MFI, mesenteric fat index*.

### Effects of Dietary DDGS Types and Levels on Serum Biochemicals and Hepatic Antioxidant Capacity

In Table 5, there were no significant interactions effects between different DDGS types and levels on serum biochemicals. Dietary DDGS types or levels had no significant effects on the serum activities of ALAT and ASAT, as well as the serum contents of TP, glucose, triglyceride, and total cholesterol either (*p* > 0.05). Hepatopancreatic GSH-Px in US-imported DDGS20 showed a significant increase compared with native DDGS20 (Table 6). However, hepatopancreatic T-AOC and T-SOD showed significant differences according to DDGS types, as native DDGS showed a significant increase compared with US-imported DDGS, while MDA showed no significant differences between different dietary groups (*p* > 0.05).

**Table 5:** The effects of different types and levels of dietary DDGS on serum biochemical^a^.

**Items**	**Diet treatments**						
	**DDGS20 (native)**	**DDGS30 (native)**	**DDGS20 (imported)**	**DDGS30 (imported)**	**Inclusion level**	**DDGS types**	**Interaction**
ALAT (U/L)	7.00 ± 2.00	7.00 ± 1.00	5.67 ± 0.58	7.33 ± 1.53	ns	ns	ns
ASAT (U/L)	111.50 ± 21.50	86.00 ± 11.79	103.67 ± 13.32	93.67 ± 15.95	ns	ns	ns
Total protein (g dL^−1^)	34.40 ± 0.70	32.52 ± 1.51	33.23 ± 2.12	33.80 ± 1.14	ns	ns	ns
Glucose (mmol/L)	2.22 ± 0.88	2.65 ± 0.76	3.15 ± 0.19	2.74 ± 0.55	ns	ns	ns
Triglycerides (mmol/L)	2.39 ± 0.46	2.34 ± 0.26	2.76 ± 0.42	2.58 ± 0.19	ns	ns	ns
Cholesterol (mmol/L)	6.52 ± 1.34	6.42 ± 0.15	7.58 ± 0.41	7.23 ± 0.41	ns	ns	ns

a *Values are means ± standard deviation (SD). Mean values with different letters are significantly different at (P < 0.05), ns: no significance. ALAT, aspartate aminotransferase; ASAT, alanine aminotransferase*.

**Table 6:** The effects of different types and levels of dietary DDGS on the hepatic antioxidant indices of juvenile grass carp^a^.

**Items**	**Diet treatments**						
	**DDGS20 (native)**	**DDGS30 (native)**	**DDGS20 (imported)**	**DDGS30 (imported)**	**Inclusion level**	**DDGS types**	**Interaction**
GSH-Px (U/mg prot)	87.67 ± 3.82^a^	90.74 ± 4.88^ab^	97.37 ± 1.89^c^	96.70 ± 1.79^bc^	ns	^*^	ns
T-AOC (U/mg prot)	1.58 ± 0.02^b^	1.58 ± 0.04^b^	1.51 ± 0.04^a^	1.51 ± 0.03^a^	ns	^*^	ns
T-SOD (U/mg prot)	9.68 ± 0.14^b^	9.49 ± 0.31^b^	9.10 ± 0.07^a^	9.00 ± 0.08^a^	ns	^*^	ns
MDA (nmol/mg prot)	7.15 ± 0.16	7.17 ± 0.16	7.29 ± 0.13	7.20 ± 0.17	ns	ns	ns

a Values are means ± SD. Mean values with different letters are significantly different at (p < 0.05). An asterisk (*) denotes a statistically significant difference, ns: no significance. GSH-Px, glutathione peroxidase; T-AOC, total antioxidant capacity; T-SOD, superoxide dismutase; MDA, malondialdehyde.

### Effects of Dietary DDGS Types and Levels on Whole Body and Dorsal Muscle Proximate Analysis

The composition of the body and dorsal muscle did not alter significantly (*p* > 0.05) with different dietary DDGS inclusion levels and types (Table 7). Interestingly, DDGS type affected the muscle collagen significantly, and muscle collagen significantly increased in the US-imported DDGS30 group compared with native DDGS30 (*p* < 0.05), while did not show a difference between the US-imported DDGS20 and native DDGS20 group.

**Table 7:** The effects of different types and levels of dietary DDGS on body and dorsal muscle proximate composition^a^.

**Items**	**Diet treatments**						
	**DDGS20 (native)**	**DDGS30 (native)**	**DDGS20 (imported)**	**DDGS30 (imported)**	**Inclusion level**	**DDGS types**	**Interaction**
**Whole body (%)**							
Moisture	78.20 ± 0.10	78.73 ± 0.35	78.63 ± 0.31	78.90 ± 0.36	ns	ns	ns
Protein	18.53 ± 0.06	18.20 ± 0.44	18.07 ± 0.38	17.67 ± 0.16	ns	ns	ns
Lipid	2.47 ± 0.06	2.40 ± 0.00	2.43 ± 0.06	2.37 ± 0.12	ns	ns	ns
Ash	0.67 ± 0.06	0.63 ± 0.06	0.70 ± 0.00	0.63 ± 0.06	ns	ns	ns
**Dorsal muscle (%)**							
Moisture	77.70 ± 0.69	78.67 ± 0.35	78.63 ± 1.04	79.23 ± 0.06	ns	ns	ns
Protein	17.67 ± 0.49	17.43 ± 0.25	17.43 ± 0.83	16.97 ± 0.06	ns	ns	ns
Lipid	3.70 ± 0.17	3.47 ± 0.12	3.63 ± 0.15	3.43 ± 0.06	ns	ns	ns
Raw muscle collagen (g/kg)	1.56 ± 0.04^ab^	1.51 ± 0.02^a^	1.56 ± 0.07^ab^	1.65 ± 0.04^b^	ns	^*^	ns

a Values are means ± SD. Mean values with different letters are significantly different at (p < 0.05). An asterisk (*) denotes a statistically significant difference.

### Effects of Dietary DDGS Types and Levels on Cooked Muscle Texture

As shown in Table 8, the cooking loss did not show any significant difference between dietary treatments (*p* > 0.05). Both DDGS inclusion level and DDGS type affected the cooked muscle texture indices, such as muscle hardness, gumminess, and chewiness with a significant interaction, which all showed a significant increase (*p* < 0.05) in the native DDGS groups compared with the US-imported DDGS groups. Furthermore, the US-imported DDGS30 showed the lowest values. With regard to springiness, a significant interaction between inclusion level and DDGS types was observed, and a significant increase (*p* < 0.05) in native DDGS20 was observed compared with other native DDGS or US-imported DDGS groups. Cohesiveness and resilience were significantly affected by DDGS types, which were significantly higher (*p* < 0.05) in native DDGS groups compared with US-imported groups, but did not show significant changes (*p* > 0.05) between increasing DDGS levels (native or US-imported).

**Table 8:** The effects of different types and levels of dietary DDGS on cooked muscle texture^a^.

**Items**	**Diet treatments**						
	**DDGS20 (native)**	**DDGS30 (native)**	**DDGS20 (imported)**	**DDGS30 (imported)**	**Inclusion level**	**DDGS types**	**Interaction**
Cooking loss (%)	20.10 ± 0.10	20.75 ± 0.85	20.07 ± 0.31	20.03 ± 0.45	ns	ns	ns
Hardness (g)	2009.72 ± 79.04^c^	1949.08 ± 8.76^c^	1270.60 ± 18.84^b^	1076.13 ± 15.34^a^	^*^	^*^	^*^
Gumminess	877.15 ± 9.83^c^	877.51 ± 7.90^c^	442.31 ± 3.41^b^	365.39 ± 2.68^a^	^*^	^*^	^*^
Chewiness (g)	409.56 ± 7.59^c^	422.47 ± 10.44^c^	206.64 ± 5.66^b^	184.59 ± 4.54^a^	^*^	^*^	^*^
Springiness	0.45 ± 0.01^c^	0.41 ± 0.00^b^	0.38 ± 0.01^a^	0.40 ± 0.00^b^	ns	^*^	^*^
Cohesiveness	0.40 ± 0.00^b^	0.39 ± 0.01^b^	0.35 ± 0.01^a^	0.36 ± 0.00^a^	ns	^*^	ns
Resilience (g/s)	0.10 ± 0.00^c^	0.09 ± 0.00^b^	0.07 ± 0.00^a^	0.07 ± 0.00^a^	ns	^*^	ns

a Values are means ± SD. Mean values with different letters are significantly different at (p < 0.05). An asterisk (*) denotes a statistically significant difference.

### Effects of Dietary DDGS Types and Levels on Hepatopancreas and Muscle Morphology

**Figure 1:**
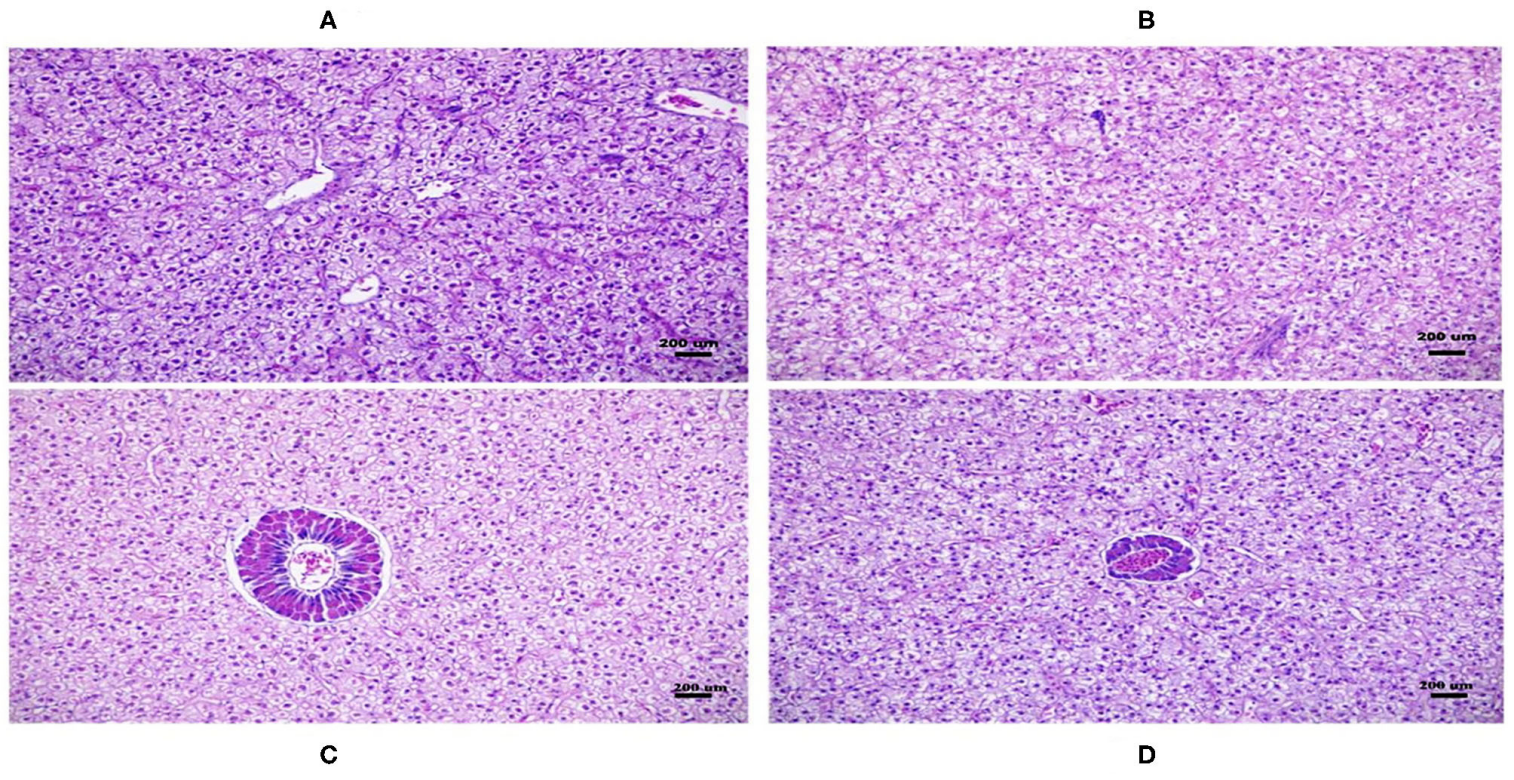
Hepatopancreatic cross-sections of the fish from the native and imported dried distillers grains with solubles (DDGS) groups stained with hematoxylin–eosin (H&E) (200 ×). Hepatopancreatic histology of native DDGS20 and DDGS30 **(A,B)** as well as imported DDGS20 and DDGS30 **(C,D)** groups showed normal hepatocyte without obvious swelling or atrophy.

**Figure 2:**
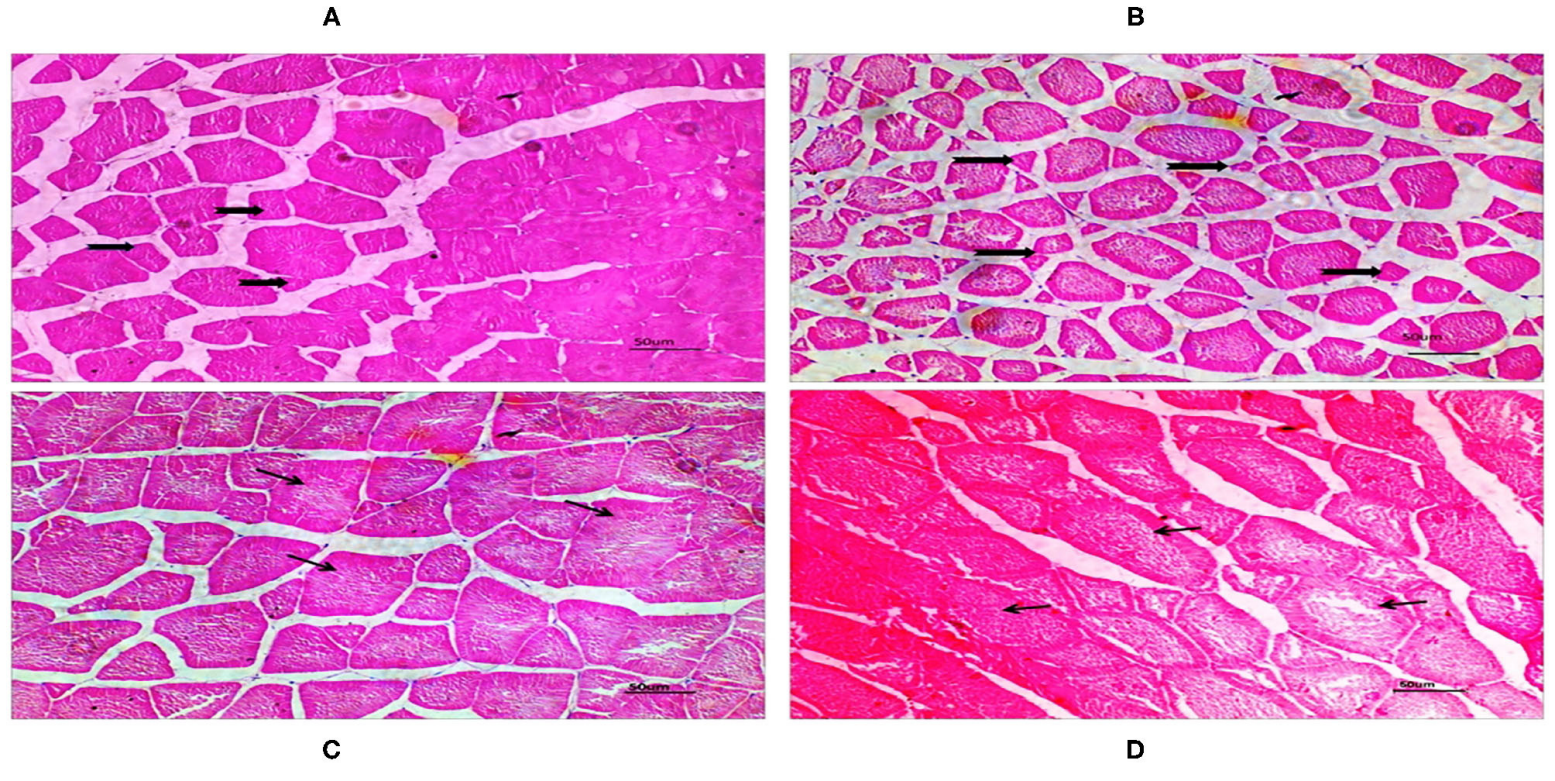
The cross-sections of dorsal muscle in juvenile grass carp with H&E (2001×). Native DDGS20 and DDGS30 groups **(A,B)** showed increased muscle fiber density, while small fiber diameter. US-imported DDGS20 and DDGS30 groups **(C,D)** showed opposite trend.

There was no difference in hepatopancreatic histology between the experimental groups. Both the native DDGS and imported DDGS groups showed normal hepatocytes without obvious swelling or atrophy (Figure 1). As shown in Table 9 and Figure 2, the muscle fiber diameter significantly decreased (*p* < 0.05) in the native DDGS groups compared with the US-imported DDGS groups. On the contrary, groups containing US-imported DDGS had significantly lower fiber density than groups including native DDGS (*p* < 0.05). Furthermore, US-imported DDGS30 showed the lowest fiber density value.

**Table 9:** The effects of different types and levels of dietary DDGS on muscle cellularity^a^.

**Items**	**Diet treatments**						
	**DDGS20 (native)**	**DDGS30 (native)**	**DDGS20 (imported)**	**DDGS30 (imported)**	**Inclusion level**	**DDGS types**	**Interaction**
Fiber diameter (μm)	47.97 ± 2.00^a^	46.72 ± 2.42^a^	57.77 ± 6.77^b^	60.69 ± 2.00^b^	ns	^*^	ns
Fiber density (N/mm^2^)	262.64 ± 9.72^c^	277.18 ± 5.13^c^	200.19 ± 18.51^b^	171.10 ± 21.37^a^	ns	^*^	^*^

a Values are means ± SD. Mean values with different letters are significantly different at (p < 0.05). An asterisk (^*^) denotes a statistically significant difference.

### Effects of Dietary DDGS Types and Levels on Muscle-Related Genes Expression

**Figure 3:**
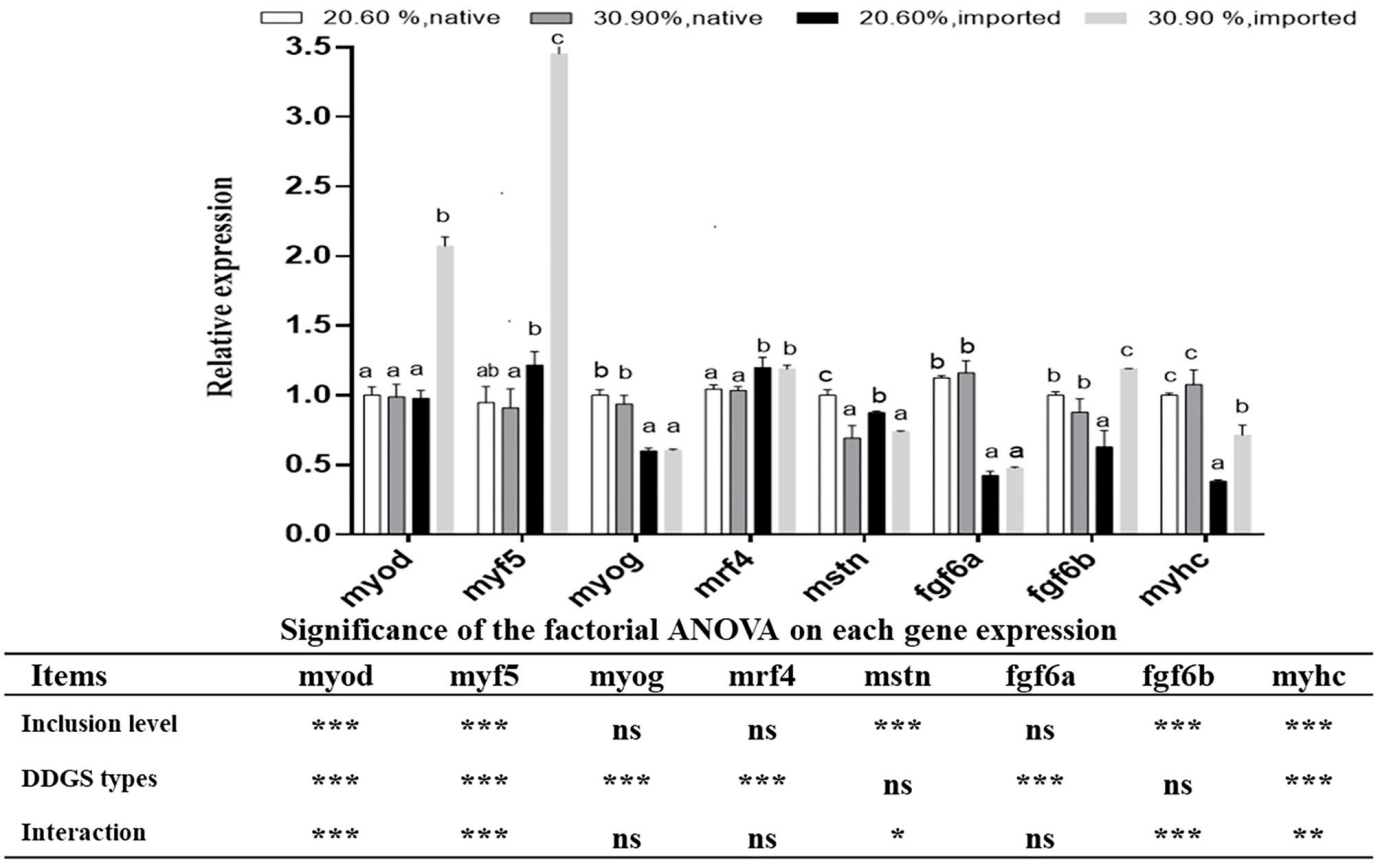
Muscle growths related gene expressions in grass carp white muscle tissue received different DDGS levels and types. Data represented the means ± SD. Bars with different letters denote significant difference between groups (*p* < 0.05). *p ≤ 0.05; **p ≤ 0.01; ***p ≤ 0.001 and ns: no significance, which mean the effect of the DDGS type and level as well as their interaction on each gene expression by the factorial ANOVA.

There were significant interaction effects between DDGS types and levels on the gene expression of *myod, myf5, mstn, fgf6b*, and *myhc*. Both *myod* and *myf5* showed significant upregulation in the US-imported DDGS30 group compared with other DDGS dietary groups (*p* < 0.05). The relative expression of *mstn* significantly decreased with increasing DDGS levels. Moreover, DDGS20 showed the highest level (*p* < 0.05). The relative expression of *fgf6b* in the US-imported DDGS30 showed significant upregulation compared with other groups. Moreover, US-imported DDGS20 showed the lowest level. The relative expression of *myhc* in the native DDGS groups showed significant upregulation compared with US-imported DDGS groups (*p* < 0.05). Furthermore, the US-imported DDGS20 showed the lowest level. DDGS types significantly affect gene expression of *myog, Mrf4*, and *fgf6a*. The expression of *myog* and *fgf6a* showed significant up-regulation in native DDGS-containing groups compared with US-imported DDGS groups (*p* < 0.05), while *mrf4* expression showed the opposite trend (Figure 3).

## Discussion

The US-imported DDGS30 group had the greatest FBW, SGR, and FE values in this investigation, showing that the growth boost was due to the better feed utilization at the appropriate DDGS inclusion level and type. This infers that the biological value of US-imported DDGS as an alternative protein source was higher than that of native DDGS, which might be related to the higher crude fiber content in Chinese native DDGS compared with US-imported DDGS, including higher acid detergent fiber (ADF) that reduced its feeding value (32). Furthermore, Chinese DDGS from different resources compared with US-imported DDGS had lower lysine, while contained similar nutrient levels (32), which might be another explanation to our findings. In this study, body and filet composition were not affected by the different levels and types of DDGS, which was in agreement with the results that the inclusion of up to 30% DDGS to European catfish (*Silurus glanis*) diet did not affect the growth parameters, nutrient utilization, biometric indices, and carcass composition (33).

There was no available literature citing the comparative effects of native and US-imported DDGS on serum biochemicals. In addition, the lack of serum biochemical reference data makes it difficult to compare the results with other studies according to fish size, species, and environmental factors (34). Serum ALAT and ASAT are important indicators that reflect liver function. A significant increase in serum ASAT and ALAT activities may indicate impaired liver function (35). In this study, serum ALAT, ASAT, TP, glucose, triglyceride, and total cholesterol levels were not significantly affected by DDGS groups. Furthermore, we found no histological changes in hepatocytes structure between the experimental groups. This is consistent with the finding that DDGS is not responsible for the observed histological changes in the absence of ANFs (36). Similarly, it was reported that plasma biochemical parameters did not show differences up to 30% DDGS dietary inclusion in European catfish (33). In contrast, serum constituents, such as triglycerides and total cholesterol increased with an increasing DDGS dietary substitution level up to 750 g/kg in laying hens (37). This means that metabolic difference exist between the aquatic and terrestrial animals.

To date, there is relatively limited information available regarding the effects of corn DDGS on the antioxidant status of grass carp. A previous study showed that dietary DDGS inclusion levels up to 30.9% decreased hepatopancreatic T-SOD, possibly due to the oxidative stress caused by higher PUFA contents in DDGS (27). Moreover, it was concluded that dietary DDGS decreased GSH and T-SOD values in juvenile grass carp (26). In the present study, hepatopancreatic GSH-Px increased in the US-imported DDGS20 group compared with the native DDGS20 group, while hepatopancreatic T-AOC and T-SOD decreased in the US-imported DDGS group compared with the native DDGS. Similarly, it was shown that the inclusion of DDGS up to 25% did not cause lipid peroxidation but increased GSH content and GSH-Px activity in the liver of broiler (38). In addition, it was reported that the antioxidant enzyme activity of GSH-Px was enhanced, to offset any oxidative stress from the dietary PUFAs when more than 18% dietary inclusion of corn DDGS in laying ducks (39). In the present study, the anti-oxidative activity of T-SOD and T-AOC decreased in US-imported DDGS containing groups that resulted in negative effects on the antioxidant system of juvenile grass carp. The mechanism of this negative influence on antioxidant enzyme activities should be elucidated in the future.

Texture profile, muscle cellularity, and gene expression were used to assess the flesh quality and skeletal muscle growth of grass carp. Changes in white muscular growth, which is caused by changes in both muscle hypertrophy and hyperplasia, account for the majority of changes in somatic growth in fish. This study confirms the plasticity of the white muscle growth dynamics of fish, which has been demonstrated to be influenced by dietary level or nutrient supply, such as dietary lipid (40, 41). The somatic growth increase observed in the grass carp fed with the US-imported DDGS30 diet was linked to the significant changes in the growth dynamics of white muscle, increasing the diameter of white muscle fibers, whereas decreasing density compared with native DDGS. These findings point to the prevalence of hypertrophic growth by increasing fiber diameter in fish received US-imported DDGS instead of hyperplasia. Likewise, it was reported that more rapid fiber enlargement occurs in fast-growing rainbow trout than in slow-growing ones (42).

The texture is a crucial marker of fish quality. Among textural criteria, filet hardness has a significant impact on the acceptability of filets (15). According to this study, the texture profile, such as hardness, gumminess, chewiness, cohesiveness, and resilience of cooked muscle in the native DDGS groups increased significantly compared with the US-imported DDGS groups, as US-imported DDGS30 showed the lowest value, which indicated the reverse effect of imported DDGS on the filet quality of grass carp. The use of DDGS in the diets affected meat quality has gained attention. It was reported that DDGS up to 15% in broiler diets tended to reduce the hardness of the breast and thigh, however the effect was not significant (43). Our previous studies regarding the effect of dietary DDGS on grass carp texture reported that dietary DDGS inclusion produced a softer cooked muscle texture (26, 27). Furthermore, texture and other flesh quality traits are influenced by muscle cellularity and connective tissue matrix (44). In general, fibers in species with a harder texture are often smaller than those in species with a softer texture (20, 45, 46). A previous study on Atlantic salmon (*Salmo salar*, L.) demonstrated a positive correlation between the muscle fiber density and firmness of smoked filet (47). It was reported that filets with low stiffness were probably associated with small muscle fiber size, suggesting a prevalence of hyperplastic growth in Senegalese sole (*Solea senegalensis*) fed diets with 100% plant protein (48), which is in agreement with our findings. Similarly, it was mentioned that total fish meal substitution in rainbow trout induced a decrease in the white muscle fiber diameter but resulted in smaller-sized fish (41). However, inconsistent results were reported in this area. It was reported that there was no clear correlation between texture and fiber size (49). Additionally, it was reported that the dietary carbohydrate inclusion in Olive flounder (*Paralichthys olivaceus*) caused a decrease in both fiber diameter and muscle hardness (50). These inconsistent results in the correlation between muscle fiber diameter and muscle hardness indicate that this field deserves a large scale of further investigation.

Although collagen enhances the texture of raw fish flesh, it is considered to be of minor contribution to the texture of flesh after cooking (51). As indicated in our earlier research, dietary DDGS inclusion in juvenile grass carp enhanced collagen production (27). Similarly, in this study, raw muscle collagen significantly increased in the US-imported DDGS30 group compared with native DDGS30, while this did not improve the flesh texture after cooking.

It is very important to clarify the effects of DDGS types and levels on the expression of growth-related genes in the muscle of juvenile grass carp. Except for *myog*, the expression patterns of the MRFs, such as *myod, myf5*, and *mrf4* revealed a general increasing trend in US-imported DDGS containing groups. Similarly, it was detected that *mrf4* and *myod* expression correlated negatively with the increase of dietary lipid level resulting in the reduced growth in juvenile Senegalese sole (52). In contrast, the upregulation of *myog* and *myhc* expression in the native DDGS-containing groups reflects the better capacity of muscle regeneration in these groups, which might suggest the compensatory process for the growth depression caused by higher crude fiber in the native DDGS. These results may explain why the hardness of muscle increased significantly in the native DDGS groups, which agrees with the results reported by Hu et al. (53). Moreover, our results are consistent with that the difference in body weight between treatments was contributed to the modulation of *myog* gene expression in male zebrafish fed a PP diet with low growth vs. high growth, which indicated that *myog* expression was more active, due to possible muscle renewal, in fish with a slower growth rate than those with a faster growth rate (54). *Mstn* has been reported to play a crucial role in the regulation of myogenesis in vertebrates, limiting both muscle cell hypertrophy and hyperplasia and hence restraining the skeletal muscle growth (55). Moreover, it was shown that MSTNs had a deleterious impact on grass carp muscle growth after 10% paper mulberry was added (46). The higher expression of *mstn* in the lower SGR group indicated that this gene might be the cause of the drop in growth performance in this study. *Fgf6* is implicated in both the proliferation and differentiation of the myogenic lineage by promoting myoblast proliferation (56). It was reported that *fgf6* is likely to be associated with prolonged muscle hyperplasia in trout (57). Notably, native DDGS mainly act on the expression of *fgf6a* rather than *fgf6b* to regulate hyperplasia in this study. These two genes have different expression profiles in our study, in which the lower expression of *fgf6a* in US-imported DDGS groups was positively correlated to the muscle fiber density, while *fgf6b* expression showed an unsteady trend, although it was suggested that *fgf6b* had a function in muscle hypertrophy growth regulation (58). These distinctions in the function of *fgf6* subtypes deserve more confirmation. An expression of *myhc* showed a general increasing trend in native DDGS-containing groups compared with the imported DDGS groups in this study. Similarly, previous studies reported that small-diameter white fibers produced during mosaic hyperplasia have been found to transiently express myosin heavy chain gene, which can be used as a muscle growth marker (59). However, it was reported that the depressed growth of juvenile trout fed plant protein rich diets was linked to the changes in white muscle growth dynamics (a decrease in the median diameter of muscle fiber) but not to substantial changes in MRF or MHC expression levels (41).

## Conclusions

The US-imported DDGS30 had a beneficial effect on the growth of juvenile grass carp *via* regulating genes expression involved in myogenesis and hypertrophy, the formation of collagen, but had negative impacts on antioxidant capacity and cooked muscle texture.

## Data Availability Statement

The original data used to support the findings of this study are available from the corresponding author upon request.

## Ethics Statement

The animal study was reviewed and approved by the Institutional Animal Care and Use Committee (IACUC) of Huazhong Agricultural University (Wuhan, China).

## Author Contributions

FRA: software, data curation, and writing—original draft preparation. FK: visualization, investigation, and software. XW, WZ, HY, and XL: project administration and investigation. QT: supervision, writing—reviewing, and editing. All authors agree to be accountable for the content of the work and contributed to the article and approved the submitted version.

## Funding

Both the National Key R&D Program of China (2019YFD0900200) and the National Natural Science Foundation of China (Grant No. 32072950) supported this work.

## Conflict of Interest

The authors declare that the research was conducted in the absence of any commercial or financial relationships that could be construed as a potential conflict of interest.

## Publisher's Note

All claims expressed in this article are solely those of the authors and do not necessarily represent those of their affiliated organizations, or those of the publisher, the editors and the reviewers. Any product that may be evaluated in this article, or claim that may be made by its manufacturer, is not guaranteed or endorsed by the publisher.

## References

[1] Liu KS Han J. Changes in mineral concentrations and phosphorus profile during dry-grind processing of corn into ethanol. Bioresour Technol. (2011) 102:3110–8. 10.1016/j.biortech.2010.10.070 21055925

[2] Anderson P V Kerr BJ Weber TE Ziemer CJ Shurson GC. Determination and prediction of digestible and metabolizable energy from chemical analysis of corn coproducts fed to finishing pigs. J Anim Sci. (2012) 90:1242–54. 10.2527/jas.2010-3605 22147488

[3] Shurson J. Maize dried distillers grains with solubles (DDGS)-a new alternative ingredient in aquafeeds. World Aquac. (2012) 43:54–8.

[4] Lim C Li E Klesius PH. Distiller's dried grains with solubles as an alternative protein source in diets of tilapia. Rev Aquac. (2011) 3:172–8. 10.1111/j.1753-5131.2011.01054.x

[5] Jie YZ Zhang JY Zhao LH Ma QG Ji C. The correlationship between the metabolizable energy content, chemical composition and color score in different sources of corn DDGS. J Anim Sci Biotechnol. (2013) 4:1–8. 10.1186/2049-1891-4-38 24066830 PMC3816793

[6] Jewison M Gale F. China's market for distillers dried grains and the key influences on its longer run potential. Distill Dried Grains Mark Usage Trends. (2013) 129–58.

[7] Fabiosa JF Hansen J Matthey H Pan S Tuan F. Assessing China' s Potential Import Demand for Distillers Dried Grain : Implications for Grain Trade. Center for Agricultural Rural Development, Lowa State University, Ames, United States (2009). Available online at: https://citeseerx.ist.psu.edu/viewdoc/download?doi=10.1.1.482.7824&rep=rep1&type=pdf

[8] Fastinger ND Latshaw JD Mahan DC. Amino acid availability and true metabolizable energy content of corn distillers dried grains with solubles in adult cecectomized roosters. Poult Sci. (2006) 85:1212–6. 10.1093/ps/85.7.1212 16830861

[9] Kim BG Kil DY Zhang Y Stein HH. Concentrations of analyzed or reactive lysine, but not crude protein, may predict the concentration of digestible lysine in distillers dried grains with solubles fed to pigs. J Anim Sci. (2012) 90:3798–808. 10.2527/jas.2011-4692 22585804

[10] Soares JA Stein HH Singh V Shurson GS Pettigrew JE. Amino acid digestibility of corn distillers dried grains with solubles, liquid condensed solubles, pulse dried thin stillage, and syrup balls fed to growing pigs. J Anim Sci. (2012) 90:1255–61. 10.2527/jas.2010-3691 22064734

[11] Stein HH Shurson GC. Board-invited review: the use and application of distillers dried grains with solubles in swine diets. J Anim Sci. (2009) 87:1292–303. 10.2527/jas.2008-1290 19028847

[12] Li P Li DF Zhang HY Li ZC Zhao PF Zeng ZK. Determination and prediction of energy values in corn distillers dried grains with solubles sources with varying oil content for growing pigs. J Anim Sci. (2015) 93:3458–70. 10.2527/jas.2014-8782 26440015

[13] Ergul T Martinez-Amezcua C Parsons CM Walters B Brannon J Noll SL. Amino acid digestibility in corn distillers dried grains with solubles. Poult Sci. (2003) 82 (Suppl. 1):70.

[14] USGC. Precision DDGS Nutrition. 4th ed. Washington, DC: USGC (2018). p. 376.

[15] Larsson T Mørkøre T Kolstad K Østbye TK Afanasyev S Krasnov A. Gene expression profiling of soft and firm Atlantic salmon fillet. PLoS ONE. (2012) 7:e39219. 10.1371/journal.pone.0039219 22745718 PMC3379969

[16] Cheng JH Sun DW Han Z Zeng XA. Texture and structure measurements and analyses for evaluation of fish and fillet freshness quality: a review. Compr Rev Food Sci Food Saf. (2014) 13:52–61. 10.1111/1541-4337.12043 33412693

[17] Fuentes A Fernández-Segovia I Serra JA Barat JM. Comparison of wild and cultured sea bass (Dicentrarchus labrax) quality. Food Chem. (2010) 119:1514–18. 10.1016/j.foodchem.2009.09.036

[18] Khan N Qureshi NA Nasir M Rasool F Iqbal KJ. Effect of artificial diet and culture systems on sensory quality of fried fish flesh of indian major carps. Pak J Zool. (2011) 43:1177–82.

[19] Moreno HM Montero MP Gómez-Guillén MC Fernández-Martín F Mørkøre T Borderías J. Collagen characteristics of farmed Atlantic salmon with firm and soft fillet texture. Food Chem. (2012) 134:678–85. 10.1016/j.foodchem.2012.02.160 23107678

[20] Periago MJ Ayala MD López-Albors O Abdel I Martínez C García-Alcázar A. Muscle cellularity and flesh quality of wild and farmed sea bass, *Dicentrarchus labrax* L. Aquaculture. (2005) 249:175–88. 10.1016/j.aquaculture.2005.02.047

[21] Rowlerson A Veggetti A. Cellular mechanisms of post-embryonic muscle growth in aquaculture species. Fish Physiol. (2001) 18:103–40. 10.1016/S1546-5098(01)18006-4

[22] Asaduzzaman M Ikeda D Abol-Munafi AB Bulbul M Ali ME Kinoshita S. Dietary supplementation of inosine monophosphate promotes cellular growth of muscle and upregulates growth-related gene expression in Nile tilapia *Oreochromis niloticus*. Aquaculture. (2017) 468:297–306. 10.1016/j.aquaculture.2016.10.033

[23] Seiliez I Sabin N Gabillard JC. Myostatin inhibits proliferation but not differentiation of trout myoblasts. Mol Cell Endocrinol. (2012) 351:220–6. 10.1016/j.mce.2011.12.011 22209759

[24] De-Santis C Jerry DR. Candidate growth genes in finfish - where should we be looking? Aquaculture. (2007) 272:22–38. 10.1016/j.aquaculture.2007.08.036

[25] Lin D. Grass carp, Ctenopharyngodon idella. In: Handbook of Nutrient Requirements of Finfish. Boca Raton, FL: CRC Press (1991). p. 89–96.

[26] Kong F Abouel Azm FR Tan Q Yu H Yao J Luo Z. Effects of replacement of dietary cotton meal by distiller's dried grains with solubles (DDGS) on growth performance, muscle texture, health and expression of muscle-related genes in grass carp (*Ctenopharyngodon idellus*). Aquac Nutr. (2021) 27:1255–66.? 10.1111/anu.13266

[27] Abouel Azm FR Kong F Tan Q Zhu Y Yu H Yao J. Effects of replacement of dietary rapeseed meal by distiller's dried grains with solubles (DDGS) on growth performance, muscle texture, health and expression of muscle-related genes in grass carp (*Ctenopharyngodon idellus*). Aquaculture. (2021) 533:736169. 10.1016/j.aquaculture.2020.736169

[28] AOAC. Official Methods of Analysis of AOAC International. 16th ed. Gaithersburg, MD: Association of Official Analytical Chemists (1995).

[29] Wongthahan P Thawornchinsombut S. Quality improvement of reduced-salt, phosphate-free fish patties from processed by-products of nile tilapia using textural additives and bioactive rice bran compounds. J Texture Stud. (2015) 46:240–53. 10.1111/jtxs.12122

[30] Bourne MC. Texture profile analysis. Food Technol. (1978) 32:62–6.?

[31] Pfaffl MW. A new mathematical model for relative quantification in real-time RT-PCR. Nucleic Acids Res. (2001) 29:e45. 10.1093/nar/29.9.e45 11328886 PMC55695

[32] Xue PC Dong B Zang JJ Zhu ZP Gong LM. Energy and standardized ileal amino acid digestibilities of Chinese distillers dried grains, produced from different regions and grains fed to growing pigs. Asian-Australasian J Anim Sci. (2012) 25:104–113. 10.5713/ajas.2011.11052 25049485 PMC4092930

[33] Sándor ZJ Révész N Varga D Tóth F Ardó L Gyalog G. Nutritional and economic benefits of using DDGS (distiller' dried grains soluble) as feed ingredient in common carp semi-intensive pond culture. Aquac Rep. (2021) 21:100819. 10.1016/j.aqrep.2021.100819

[34] Cheng ZJ Hardy RW Blair M. Effects of supplementing methionine hydroxy analogue in soybean meal and distiller's dried grain-based diets on the performance and nutrient retention of rainbow trout [Oncorhynchus mykiss (Walbaum)]. Aquac Res. (2003) 34:1303–10. 10.1046/j.1365-2109.2003.00940.x

[35] Wang XF Li XQ Leng XJ Shan LL Zhao JX Wang YT. Effects of dietary cottonseed meal level on the growth, hematological indices, liver and gonad histology of juvenile common carp (Cyprinus carpio). Aquaculture. (2014) 428–429:79–87. 10.1016/j.aquaculture.2014.02.040

[36] Révész N Havasi M Lefler KK Hegyi Á Ardó L Sándor Z. Protein replacement with dried distiller's grain with solubles (DDGS) in practical diet of common carp (cyprinus carpio). AACL Bioflux. (2019) 12:1174–88.

[37] Abd El-Hack ME Chaudhry MT Mahrose KM Noreldin A Emam M Alagawany M. The efficacy of using exogenous enzymes cocktail on production, egg quality, egg nutrients and blood metabolites of laying hens fed distiller's dried grains with solubles. J Anim Physiol Anim Nutr. (2018) 102:e726–35. 10.1111/jpn.12825 28990277

[38] Heincinger M Balogh K Fébel H Erdélyi M Mézes M. Effect of diets with different inclusion levels of distillers dried grain with solubles combined with lysine and methionine supplementation on the lipid peroxidation and glutathione status of chickens. Acta Vet Hung. (2011) 59:195–204. 10.1556/avet.2011.005 21665573

[39] Ruan D Fouad AM Fan QL Chen W Xia WG Wang S. Effects of corn dried distillers' grains with solubles on performance, egg quality, yolk fatty acid composition and oxidative status in laying ducks. Poult Sci. (2018) 97:568–77. 10.3382/ps/pex331 29211867

[40] Fauconneau B Andre S Chmaitilly J Le Bail PY Krieg F Kaushik SJ. Control of skeletal muscle fibres and adipose cells size in the flesh of rainbow trout. J Fish Biol. (1997) 50:296–314. 10.1111/j.1095-8649.1997.tb01360.x

[41] Alami-Durante H Wrutniak-Cabello C Kaushik SJ Médale F. Skeletal muscle cellularity and expression of myogenic regulatory factors and myosin heavy chains in rainbow trout (*Oncorhynchus mykiss*): effects of changes in dietary plant protein sources and amino acid profiles. Comp Biochem Physiol Mol Integr Physiol. (2010) 156:561–8. 10.1016/j.cbpa.2010.04.015 20434580

[42] Kiessling A Storebakken T Åsgård T Kiessling KH. Changes in the structure and function of the epaxial muscle of rainbow trout (*Oncorhynchus mykiss*) in relation to ration and age. I. Growth dynamics. Aquaculture. (1991) 93:335–56. 10.1016/0044-8486(91)90225-V

[43] Choi HS Lee HL Shin MH Jo C Lee SK Lee BD. Nutritive and economic values of corn distiller's dried grains with solubles in broiler diets. Asian Australasian J Anim Sci. (2008) 21:414–9. 10.5713/ajas.2008.70067

[44] Johnston IA. Muscle development and growth: potential implications for flesh quality in fish. Aquaculture. (1999) 177:99–115. 10.1016/S0044-8486(99)00072-1

[45] Hatae K Yoshimatsu F Matsumoto JJ. Role of muscle fibers in contributing firmness of cooked fish. J Food Sci. (1990) 55:693–6. 10.1111/j.1365-2621.1990.tb05208.x

[46] Tang T Bai J Ao Z Wei Z Hu Y Liu S. Effects of dietary paper mulberry (Broussonetia papyrifera) on growth performance and muscle quality of grass carp (*Ctenopharyngodon idella*). Animals. (2021) 11:1655. 10.3390/ani11061655 34199491 PMC8227960

[47] Johnston IA Alderson R Sandham C Dingwall A Mitchell D Selkirk C. Muscle fibre density in relation to the colour and texture of smoked Atlantic salmon (*Salmo salar* L.). Aquaculture. (2000) 189:335–49. 10.1016/S0044-8486(00)00373-2

[48] Valente LMP Cabral EM Sousa V Cunha LM Fernandes JMO. Plant protein blends in diets for Senegalese sole affect skeletal muscle growth, flesh texture and the expression of related genes. Aquaculture. (2016) 453:77–85. 10.1016/j.aquaculture.2015.11.034

[49] Ayala MD Abdel I Santaella M Martínez C Periago MJ Gil F. Muscle tissue structural changes and texture development in sea bream, *Sparus aurata* L., during post-mortem storage. LWT Food Sci Technol. (2010) 43:465–75. 10.1016/j.lwt.2009.08.023

[50] Liu J Deng K Pan M Liu G Wu J Yang M. Dietary carbohydrates influence muscle texture of olive flounder *Paralichthys olivaceus* through impacting mitochondria function and metabolism of glycogen and protein. Sci Rep. (2020) 10:1–20. 10.1038/s41598-020-76255-3 33311521 PMC7732841

[51] Hatae K Matsumoto JJ Tobimatsn A Takeyama M. Contribution of the connective tissues on the texture difference of various fish species. Nippon Suisan Gakkaishi. (1986) 52:2001–7. 10.2331/suisan.52.2001

[52] Campos C Valente LMP Borges P Bizuayehu T Fernandes JMO. Dietary lipid levels have a remarkable impact on the expression of growth-related genes in Senegalese sole (*Solea senegalensis* Kaup). J Exp Biol. (2010) 213:200–9. 10.1242/jeb.033126 20038653

[53] Hu Y Hu Y Wu T Chu W. Effects of high dietary levels of cottonseed meal and rapeseed meal on growth performance, muscle texture, and expression of muscle-related genes in grass carp. N Am J Aquac. (2019) 81:235–41. 10.1002/naaq.10091

[54] Ulloa PE Peña AA Lizama CD Araneda C Iturra P Neira R. Growth response and expression of muscle growth-related candidate genes in adult zebrafish fed plant and fishmeal protein-based diets. Zebrafish. (2013) 10:99–109. 10.1089/zeb.2012.0823 23590402

[55] Lee SJ McPherron AC. Regulation of myostatin activity and muscle growth. Proc Natl Acad Sci USA. (2001) 98:9306–11. 10.1073/pnas.151270098 11459935 PMC55416

[56] Armand AS Launay T Pariset C Della Gaspera B Charbonnier F Chanoine C. Injection of FGF6 accelerates regeneration of the soleus muscle in adult mice. Biochim Biophys Acta Mol Cell Res. (2003) 1642:97–105. 10.1016/S0167-4889(03)00103-4 12972298

[57] Rescan PY. Identification of a fibroblast growth factor 6 (FGF6) gene in a non- mammalian vertebrate: continuous expression of FGF6 accompanies muscle fiber hyperplasia. Biochim Biophys Acta Gene Struct Expr. (1998) 1443:305–14. 10.1016/S0167-4781(98)00233-4 9878802

[58] Xu Y Tan Q Hu P Yao J. Characterization and expression analysis of FGF6 (fibroblast growth factor 6) genes of grass carp (Ctenopharyngodon idellus) reveal their regulation on muscle growth. Fish Physiol Biochem. (2019) 45:1649–62. 10.1007/s10695-019-00655-0 31140072

[59] Rescan PY. Muscle growth patterns and regulation during fish ontogeny. Gen Comp Endocrinol. (2005) 142:111–6. 10.1016/j.ygcen.2004.12.016 15862555

